# Bond Performance of GFRP Bars in Glass and Basalt Fiber-Reinforced Geopolymer Concrete Under Hinged Beam Tests

**DOI:** 10.3390/ma18030498

**Published:** 2025-01-22

**Authors:** Duygu Ertürkmen, Hüsamettin Ürünveren, Ahmet Beycioğlu, Nabi Ibadov, Hüseyin Yılmaz Aruntaş, Andrzej Garbacz

**Affiliations:** 1 Department of Civil Engineering, Faculty of Engineering, Mersin University, Mersin 33343, Turkey; derturkmen@mersin.edu.tr; 2 Department of Civil Engineering, Faculty of Engineering, Adana Alparslan Türkeş Science and Technology University, Adana 01250, Turkey; hurunveren@atu.edu.tr (H.Ü.); abeycioglu@atu.edu.tr (A.B.); 3 Faculty of Civil Engineering, Department of Production Engineering and Management in Construction, Warsaw University of Technology, Al. Armii Ludowej 16, 00-637 Warszawa, Poland; nabi.ibadov@pw.edu.pl; 4 Department of Civil Engineering, Faculty of Technology, Gazi University, Ankara 06500, Turkey; aruntas@gazi.edu.tr; 5 Faculty of Civil Engineering, Department of Building Materials Engineering, Warsaw University of Technology, Al. Armii Ludowej 16, PL 00637 Warsaw, Poland

**Keywords:** GFRP bars, geopolymer concrete, chopped basalt fiber, chopped glass fiber, bond stress, hinged beam test

## Abstract

In recent years, researchers have focused on the usability of fiber-reinforced polymer (FRP) bars due to their lightweight, corrosion-resistant, and eco-friendly characteristics. Geopolymers, as low-carbon alternatives to traditional binders, aim to reduce CO_2_ emissions in concrete production. The bond strength between FRP bars and concrete is critical for the load-bearing capacity and deformation characteristics of reinforced elements. The objectives of this work are to investigate the bond performance of GFRP bars in chopped glass and basalt fiber-added geopolymer concrete using hinged beam tests. Since the hinged beam test accurately represents the behavior of real bending elements, this test method was selected as a main bonding test. Initially, three geopolymer mixtures with Ms modulus values of 1.2, 1.3, and 1.4 were prepared and tested. The mixture with a modulus of 1.2 Ms, achieving a compressive strength of 56.53 MPa, a flexural strength of 3.54 MPa, and a flow diameter of 57 cm, was chosen for beam production due to its optimal workability and strength. After mechanical and workability tests, SEM analysis was performed to evaluate its internal structure. For evaluating the bond performance of GFRP bars, 12 geopolymer beam specimens were prepared, incorporating varying fiber types (chopped glass fiber or basalt fiber) and embedment lengths (5 Ø or 20 Ø). Hinged beam tests revealed that the bond strengths of glass and basalt fiber-added mixtures were up to 49% and 37% higher than that of the control geopolymer concrete, respectively. It was concluded that incorporating fibers positively influenced the bond between geopolymer concrete and GFRP bars, with glass fibers proving more effective than basalt fibers. These findings enhance the understanding of bond mechanisms between GFRP bars and geopolymer concrete, emphasizing their potential for sustainable and durable construction in both industrial and scientific applications.

## 1. Introduction

Fibers are materials with different mechanical and physical properties that are added to concrete mix in certain amounts to improve the low tensile strength of concrete and increase the ductility of concrete. Apart from steel fibers, which are commonly used for this purpose today, there are various types of fibers available, including carbon, glass, basalt, aramid, and polypropylene [1,2,3]. The important thing for the designer is, of course, to be able to choose the most economical fiber type and the best one in terms of performance compared to the others.

It has been determined that the tensile strength of basalt fibers is approximately twice the tensile strength of glass fibers, and the modulus of elasticity of basalt fibers is approximately 15–30% higher than that of glass fibers. It is also stated that basalt fiber composites can be used instead of steel fiber in case of corrosion [4]. However, fibers should not be used at too high a ratio in mixtures. According to research, it has been reported that the modulus of elasticity values decreases with increasing fiber volume in the mixture, and at the same time, the workability is negatively affected [5,6]. In general, it can be said that there is a significant improvement in the tensile strength and flexural strength values of basalt fiber concretes compared to non-fiber concretes [7]. In a study investigating the effects of steel fibers on the bond strength between high-strength concrete and fiber-reinforced polymer bars, it was determined that the bond strength increased as the amount of fibers in the mixture increased [8]. Another study found that the bond strength of geopolymer concrete is enhanced with the addition of fibers [9].

Steel rebars in reinforced concrete elements of buildings may corrode over time due to various factors. Corroded rebars may no longer perform as expected under service conditions, and intervention becomes very difficult and costly. Corrosion-resistant, lightweight fiber-reinforced polymer (FRP) bars with high tensile strength and easy processing are a good choice for this purpose. However, despite these advantages, FRP bars also have disadvantages, such as higher initial costs, brittle tensile behavior, low compressive strength, low modulus of elasticity values, and limited production when compared to steel rebars [10]. The bond strength of FRP bars with concrete is much lower than the related values of steel rebars [11]. The bonding behavior of FRP bars with concrete is influenced by various parameters, including fiber type, modulus of elasticity, surface texture (surface characteristic) of the rebar, reinforcement position, embedment length of the bar in concrete, bar diameter, compressive strength of concrete, fiber contribution, concrete cover, and bar spacing [12,13].

The literature on test methods other than the direct pull-out test is limited, and data on these methods are insufficient.

There are four types of fiber-reinforced bars that can be used in structural engineering applications: carbon fiber-reinforced polymer (CFRP) bars, basalt fiber-reinforced polymer (BFRP) bars, glass fiber-reinforced polymer (GFRP) bars, and aramid fiber-reinforced polymer (AFRP) bars. Carbon fiber bars have a high tensile strength and high modulus of elasticity, but they are not suitable for high-temperature applications, and they are expensive. They cost 10 to 30 times more than glass fiber bars. BFRP has a similar chemical composition to GFRP but possesses better strength properties. BFRPs are a good choice to use in concrete, bridges, and coastal structures due to their resistance to alkaline, acidic, and salt attacks. They also have higher shear strength than other types and high-temperature resistance. While the price of BFRPs is higher than GFRPs, it is lower than that of AFRPs or CFRPs. Recently, an application of hybrid (HFRP) bars was also considered to increase mechanical properties [14,15].

GFRP bars with alkaline properties have started to be frequently preferred in the construction industry. GFRP bars are lightweight reinforcements with a long service life and corrosion resistance. Their tensile strength is higher than steel rebar, but their modulus of elasticity is lower. The material exhibits a linear elastic behavior until the moment of collapse, so it shows brittle behavior by breaking suddenly and cannot behave as ductile as steel rebar. However, in the design of reinforced concrete beams, with the increasing load effect, it is desired that the collapse occurs with the crushing of the concrete in the compression zone following the yielding of the reinforcement in the tension zone. However, it is important to note that GFRP bars are lighter than steel rebars, which can significantly reduce the weight of the structure. This, in turn, can decrease the potential earthquake force and resulting damage to the structure [14]. For this reason, the GFRP bar should be used together with steel rebar, especially in structures to be built in earthquake zones, and the earthquake loads that may be applied to the structure should be reduced as much as possible. It is important to note that GFRP bars have lower axial stiffness and bond compared to steel rebar, which may result in a higher number of cracks and wider crack widths in the elements. For this reason, it is recommended that GFRP bars and steel rebars should be used together to ensure more ductile behavior of the structural element [15,16].

Numerous studies have been conducted on the bond between standard concrete and steel rebar in the literature. A review of existing studies reveals that several investigations have been conducted on the bond between GFRP bars and normal and high-strength concrete [17,18,19,20,21]. Furthermore, the number of studies examining the bond between geopolymer concrete and GFRP bars is insufficient. In a study investigating the bond between GFRP bars and steel fiber concrete using a hinged beam bending test, bond stress-stripping curves of the specimens were generated by varying the surface type, diameter, and embedment length of the bar. In the study, pull-out failure occurred for all embedment lengths of 5Φ, 10Φ, and 20Φ for specimens containing GFRP bars. It was reported that all specimens reached the ultimate strength at tensile failure, and the bar surface had a significant effect on bonding [22]. In another study examining the effect of CFRP, GFRP, and steel fiber addition on the bond strength of concrete, direct pull-out tests were applied to the prepared specimens, and it was observed that steel fibers provided lower bond strength than CFRP and GFRP reinforcement [23]. For the bond strength between the GFRP bar and concrete, an equation describing the bond stress has been developed using the results of previous direct pull-out tests available in the literature so far [24]. The diameter of the GFRP bar, the thickness of the concrete cover, and the embedment length considered in the experiment had a direct effect on the bond stress values [25]. A study was conducted to investigate the impact of challenging environmental temperature conditions, such as freeze–thaw effects, on the bond strength of the GFRP bar. The study involved performing concentric and eccentric direct tensile tests on 36 concrete specimens. In the experiments, the GFRP bar’s diameter, concrete cover thickness, and applied temperature values were kept variable. It was observed that as the bar’s diameter increased, the bond strength decreased, and as the concrete thickness increased, the fracture modes of the concrete changed depending on the temperature [26].

Concrete is a fundamental component of modern infrastructure, widely used in the construction of buildings, bridges, dams, cement pavements, and other essential structures due to its versatility, strength, and durability [27].

Concrete is commonly used because of its benefits, including easy pouring and availability. However, the cement used as a binder releases a large amount of carbon dioxide during production, leading to a significant environmental impact. As a result, the search for alternative materials is becoming increasingly urgent. Low carbon and high performance have become key trends in the development of construction materials [28,29].

Due to environmental concerns and the CO_2_ emissions associated with cement production, recent literature [30,31] has increasingly explored alternative binder designs using industrial waste materials directly, rather than as substitutes for cement.

Geopolymer is a new type of binder made by the alkaline activation of industrial waste materials. As an innovative green binder material, geopolymer is formed by activating aluminosilicate-rich raw materials like blast furnace slag and fly ash with an alkali activator. The process involves the breaking of chemical bonds, such as -Si-O-Si-, -Si-O-Al-, and -Al-O-Al-, within these raw materials in a highly alkaline environment, releasing active silicon (Si) and aluminum (Al) monomers. These monomers then undergo polymerization, resulting in a three-dimensional network structure made up of SiO_4_ and AlO_4_ units interconnected by shared oxygen atoms [32].

As a result of the reaction between aluminosilicate-based fly ash and alkali activators, amorphous inorganic polymers known as ‘geopolymers’ are formed.

There has been growing interest in the development, characterization, and application of geopolymers for various uses, including sustainable construction and high-temperature-resistant manufacturing. However, geopolymers typically exhibit weak tensile strength and are prone to brittle failure. Many studies have focused on incorporating various types of fibers into geopolymers to enhance their toughness and mechanical and thermal properties [33,34].

Several studies have investigated the bond strength of geopolymer concrete compared steel rebar [35,36,37].

Bayrak et al., (2024) studied 192 pull-out specimens to investigate the factors influencing the bond-slip behavior between geopolymer and steel rebar. Key factors included curing conditions, rib angle, rebar position, bar diameter, stirrup usage, and bond length. Results showed that rib angle, rebar position, and bar diameter significantly affect bond strength. Specimens with stirrups showed greater bond improvement, especially as the bar diameter increased. Stirrup usage enhanced bond strength by 45% under ambient curing and 40% under heat curing. Curing conditions also impacted bond performance, with heat-cured samples showing improved compressive and tensile strengths. Doubling the bond length increased bond strength by 70%, and increasing bar diameter improved bond strength by up to 84%. These findings provide valuable insights into the bond behavior of geopolymers for the effective use of geopolymer-bonded rebar in construction [38].

Özdemir et al., (2025) investigated the bond performance of geopolymer concrete using pull-out tests, with experimental parameters including fiber type (basalt and glass), bar diameter (8 mm, 16 mm, and 24 mm), rebar position, and exposure to high temperatures (20 °C, 200 °C, 400 °C, 600 °C, and 800 °C). The results indicated that bar diameter is a key factor affecting bond strength. While increasing the bar diameter generally reduced bond strength, some samples showed improved bond strength with larger diameters. The bond strength exhibited significant variation with temperature, showing a strong correlation between bond strength and temperature. Samples exposed to 200 °C experienced an increase in bond strength, whereas those exposed to 600 °C and 800 °C saw a significant decrease. The study concluded that both the rebar position and fiber type play important roles in influencing the bond characteristics [39].

While there is limited research on the bond performance between geopolymer concrete and GFRP bars using hinged beam tests, previous studies have primarily utilized direct pull-out tests [40,41]. A study was conducted to examine the bond performance of metakaolin-containing geopolymer concrete with steel rebar and GFRP bars. The study reported that concrete cracking occurred in geopolymer mixtures containing steel rebar, while bar pull-out occurred in geopolymer concrete with GFRP bars. The bond strength of steel rebar was found to be higher than that of GFRP bars in normal cement concrete mixtures [41].

Although the hinged beam test accurately represents the behavior of real bending elements, it is rarely used in experimental studies in the literature and is mostly found in very recent research. In a study reported by Guo et al., (2025), the interface behavior of GFRP bars in geopolymer concrete was examined through hinged beam tests, considering factors such as concrete type (geopolymer and ordinary), concrete strength (C30, C50, and C70), surface treatment of bars (shallow thread, spiral wrap ribs, and sandblasted), bar diameters (6 mm, 10 mm, and 16 mm), and bond lengths (two, four, and eight times the bar diameter). The results demonstrated a consistent bond mechanism between both geopolymer and ordinary concrete [42].

This study investigates the bonding behavior between GFRP bars and fiber-reinforced geopolymer concrete by considering different embedment lengths. Twelve beam specimens containing basalt and glass fibers were prepared and subjected to four-point loading tests using the hinged beam test method. The study focuses on the bond behavior of the GFRP bar placed in the tension zone of the beams against the applied load. The study examines the bond strength of the beam specimens produced using a certain amount of fibers in the mixture. Two different embedment lengths of 12 mm diameter GFRP bars were used to compare the results.

## 2. Materials and Methods

### 2.1. Materials

This study experimentally investigates the bond strength between fiber-reinforced geopolymer concrete and GFRP bars using beam specimens with different embedment lengths. For this purpose, a total of 12 geopolymer concrete beam specimens were produced, using various fiber types (basalt or glass) and embedment lengths (5 Ø or 20 Ø). Four specimens contained 4 kg/m^3^ of chopped basalt fiber, another four included 4 kg/m^3^ of chopped glass fiber, and the last four were made without any fibers.

Fly ash, obtained from the Sugözü power plant located in the Yumurtalık district of Adana province, was used as an aluminosilicate powder material. Crystalline sodium hydroxide (NaOH) pellets and liquid sodium silicate (Na_2_SiO_3_) solutions taken from Demirbey Chemistry, Adana, Turkey were employed as alkaline activators in the production of geopolymer concrete. When preparing the alkali activator, heat is generated by the exothermic reaction of sodium hydroxide dissolved in liquid sodium silicate. To ensure that the temperature was suitable for concrete casting, the alkaline activator was prepared in the laboratory about 24 h before casting and allowed to cool.

In the initial stage of the study, geopolymer concrete samples with varying Ms modulus values (1.1, 1.2, 1.3) under the influence of variable curing temperatures (80 °C, 100 °C, 120 °C) were prepared for compressive strength tests. A geopolymer concrete mixture that met the required compressive strength and workability was chosen for the hinged beam bond test. The Ms modulus was calculated using Equations (1) and (2) as 1.2 at 10% Na_2_O concentration, and the aggregate–fly ash ratio was assumed to be 3/1 in the mixture calculation.

(1) $$Ms=\frac{{SiO}_{2}}{{Na}_{2}O}=\frac{\sum {SiO}_{2}\;\mathrm{by}\;\mathrm{weight}(\mathrm{from}\;\mathrm{sodium}\;\mathrm{silicate})}{{\sum Na}_{2}O\;\mathrm{by}\;\mathrm{weight}\;(\mathrm{from}\;\mathrm{sodium}\;\mathrm{hydroxide}\;\mathrm{and}\;\mathrm{sodium}\;\mathrm{silicate})}$$

(2) $${Na}_{2}O\left(\mathrm{concetration}(\%)\right)=\%FA(\mathrm{by}\;\mathrm{weight})$$

where

SiO_2_: silicon dioxide;
Na_2_O: sodium dioxide;
FA: fly ash.

The physical properties of crushed limestone aggregates and the chemical composition of the fly ash used in the mixtures are given in Table 1 and Table 2, respectively. The chemical composition of fly ash was determined by X-ray fluorescence spectrometry (XRF) analysis. Two sets of samples were taken from each aggregate type (fine and coarse), and a total of 6 tests were conducted on these samples with 3 repetitions. Standard deviations (STD) obtained from the data for the experimental results are presented below each value.

When the chemical analysis results of the fly ash are evaluated, it is seen that the Si/Al, Si/(Al + Fe), and SiO_2_/(CaO + MgO) ratios are 2.52, 1.52, and 10.52, respectively.

**Figure 1:**
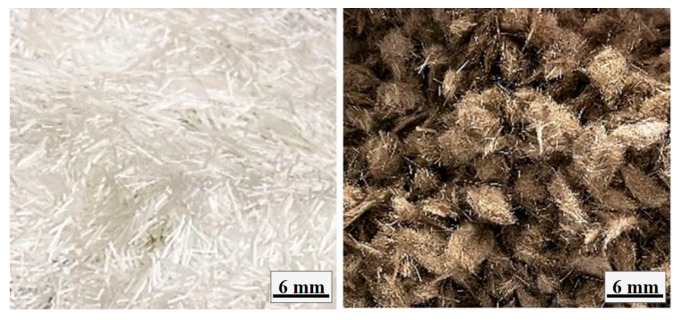
Images of chopped glass and basalt fibers.

Based on the preliminary experiments, an Ms modulus of 1.2 was chosen, and the most appropriate curing temperature was observed to be 100 °C. The amounts of materials used in the geopolymer concrete mixtures are given in Table 3. The aggregates used in the mixtures were adjusted to be 35% coarse and 65% fine aggregates. Water was added to the mixtures to ensure a saturated dry surface according to the water-absorption capacities specified in the physical properties of the aggregates (Table 1), and extra water that could change the workability properties was not used in the mixtures. The specimens were prepared for all final tests according to the proportions given in Table 3, and chopped basalt and chopped glass fibers were added to the mixtures at a volume of 4 kg/m^3^. The images of the glass and basalt fibers used in the mixtures are given in Figure 1.

All prepared specimens including hinged beams were cured in an oven at 100 °C for 24 h. The compressive and flexural tensile strengths were carried out on the samples after oven curing. Both cubic (150 × 150 × 150 mm) and prismatic (100 × 100 × 500 mm) samples were utilized for this purpose. To assess the bond performance of the geopolymer beam specimens, GFRP bars with 12 mm diameter were used in the beams. Some mechanical properties of the GFRP bars used in the study are given in Table 4.

**Figure 2:**
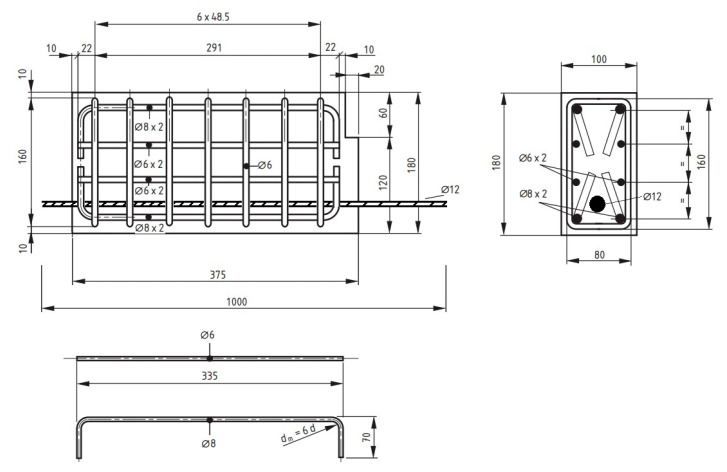
Details of the steel rebars used as stirrups in the hinged beam specimens (dimensions are given in mm).

**Figure 3:**
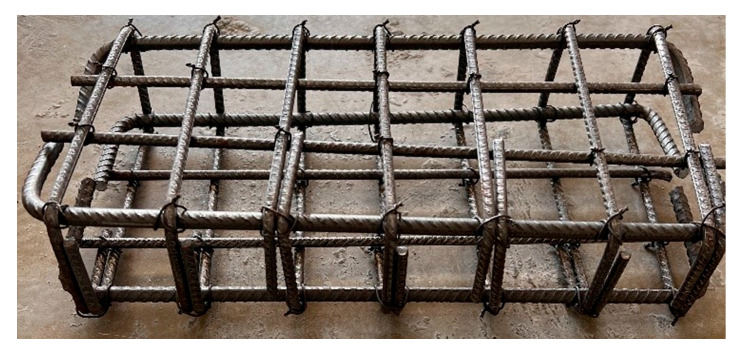
Stirrup rebars in the hinged beam specimens.

**Figure 4:**
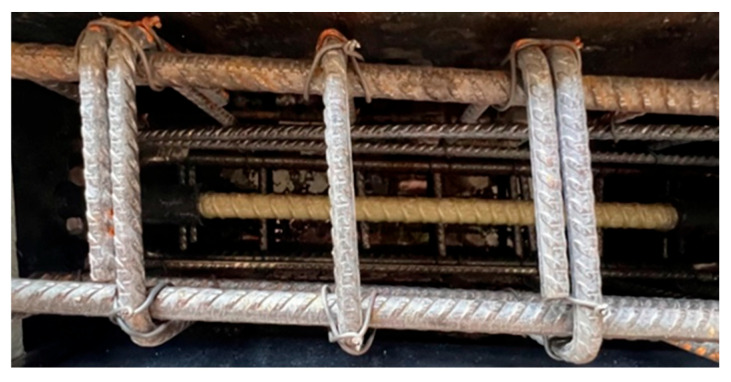
Details of an example of GFRP bar placement in the formwork.

**Figure 5:**
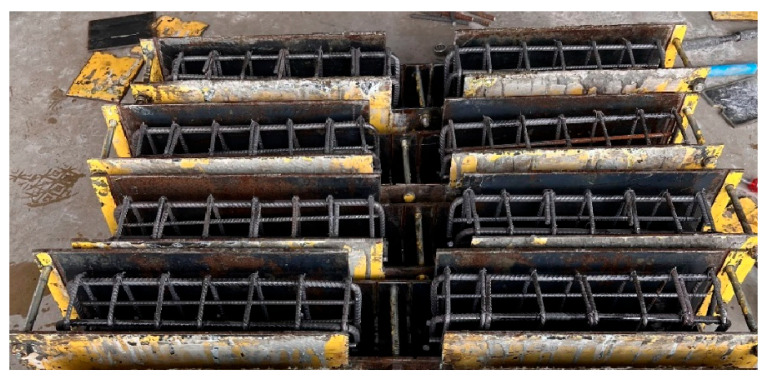
Formworks used in the preparation of hinged beam specimens.

Beam samples are generally exposed to moment cracks and/or shear cracks under bending loads. In the experimental setup used in this study, since the beam samples are empty from the middle point and transfer load with reinforcement, the risk of shear cracks during the test is quite high. It is essential to conduct the experiments without any shear cracks. To ensure the shear resistance of the beam specimens, stirrups were utilized. Figure 2 presents the details of the steel rebars used as stirrups for shear strength in the hinged beam specimens, and Figure 3, Figure 4 and Figure 5 show the application of the stirrups in the specimens. Figure 2 presents the details of the steel rebars used as stirrups for shear strength in the hinged beam specimens, and Figure 3, Figure 4 and Figure 5 show the application of the stirrups in the specimens.

Two different embedment lengths of 5 Ø and 20 Ø were considered for the bond tests on 12 mm diameter GFRP bars. The embedment length for ribbed bars in reinforced concrete was determined using Equation (3) specified in TS 500 “Reinforced Concrete Structures Design and Construction Rules” 2000 [43],

(3) $$l_{b}=(0.12\times f_{yd}\times f_{ctd}\times Ø)\geq 20Ø$$

where

*l*_*b*_: embedment length;
*f*_*yd*_: design yield strength of the reinforcement;
*f*_*ctd*_: design tensile strength of the concrete used;
Ø: reinforcement diameter.

A 20 Ø embedment length is recommended as a safe embedment length in the TS500 standard. This recommended value was used as an experimental parameter to determine whetherthe strong bonding could be observed between GFRP and geopolymer concrete without any stripping at the safe embedment length. In addition, 5 Ø was used to include a short embedment length in order to compare the bonding between GFRP and geopolymer concrete in the event of no stripping when the 20 Ø embedment length was used.

To determine the bond performance of longitudinal bars, plastic sheaths were used to limit embedment lengths. Hot silicone was applied to the limits defined by the embedment lengths to prevent concrete from penetrating between the sheaths and the bars. Figure 4 shows the details of an example of a GFRP bar placed in the formwork, and Figure 5 shows the formwork used in the experiments.

**Figure 6:**
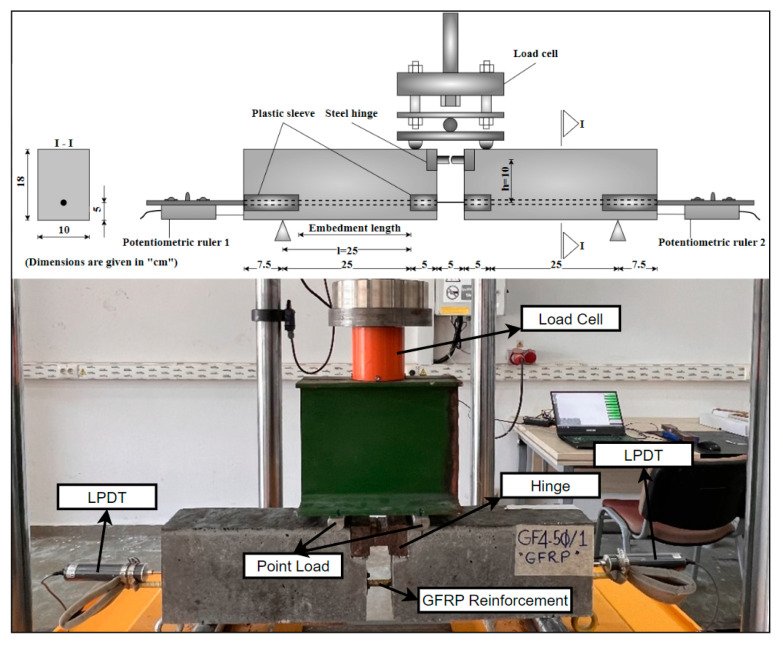
Schematic illustration of the hinged beam test setup and an image of a beam specimen before the test.

The concrete was poured into the prepared formworks in two stages, and a shaking table was used to ensure uniform compaction of the concrete. After curing in the oven for 24 h at 100 °C, the hinged beam specimens were removed from the formworks for testing. Figure 6 shows a schematic illustration of the hinged beam test setup and an image of a beam specimen prepared for the hinged beam test.

### 2.2. Methods

Since it represents the real structural behavior better than the direct tensile method, hinged beams consistent with the BS 4449:2005+A2:2009 standard [44] were used for bonding tests. The beams were prepared and placed on two supports, one fixed and one movable. They were then loaded vertically from the center point of the beam. The loads were applied to the beam at two points using a plate specially prepared for the test [44,45,46,47,48]. A four-point loading system was used in the test. The test beams were subjected to vertical loads using a hydraulic piston with a capacity of 1000 kN, at a speed of 0.5 kN/s, and the load values were determined by a load cell. Potentiometric displacement transducers (LPDTs) with an accuracy of 0.01 mm were utilized to measure the amount of stripping of the GFRP bar from the concrete. The applied loads and their respective stripping values were simultaneously recorded by a data collection system.

**Figure 7:**
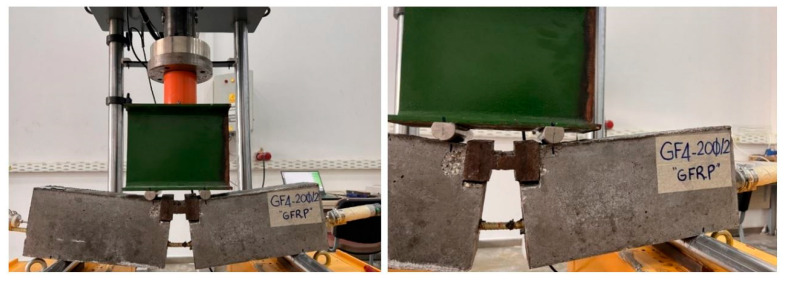
Images from the hinged beam test.

Figure 7 shows some images from the experiment. As can be seen from Figure 7, to measure the force acting on the GFRP bar accurately, a steel hinge was placed at the top center of the beam specimen.

During testing, the GFRP bar may experience different slip values at both ends due to variations in concrete composition and loadings. For safety, it is recommended to use the larger value. The equation between the load on the beam and the force on the GFRP bar can be expressed as Equation (4), since the moment at the center point of the steel hinge is zero. To determine the force (*F*) in the GFRP bar, Equation (5) can be used indirectly with the help of external load (*P*), given that *l* = 25 cm and *h* = 10 cm in the produced beams.

(4) (P×l)/2=F×h

(5) F=1.25×P

Equation (6) is obtained by ensuring that the tensile force (*F*) on the GFRP bar is in balance along the embedment length (*ℓb*), which is equal to the total bond force around the GFRP bar. This equation uses σ_s_ to represent the stress on the GFRP bar, *τ_b_* to represent the bond stress, and *ℓb* to represent the embedment length. After necessary revisions, Equation (7) can be used to calculate the bond stress (*τ_b_*) for any load value.

(6) $$\tau_{b}\times \left(\pi \times Ø\right)\times lb=A\times \sigma_{s}=\frac{\pi \times {Ø}^{2}}{4}\times \sigma_{s}$$

(7) $$\tau_{b}=\left(\sigma_{s}\times Ø\right)/4(lb)$$

The produced beams were labeled using a specific code to indicate their characteristics. The coding details for all beam specimens are presented in Table 5.

**Figure 8:**
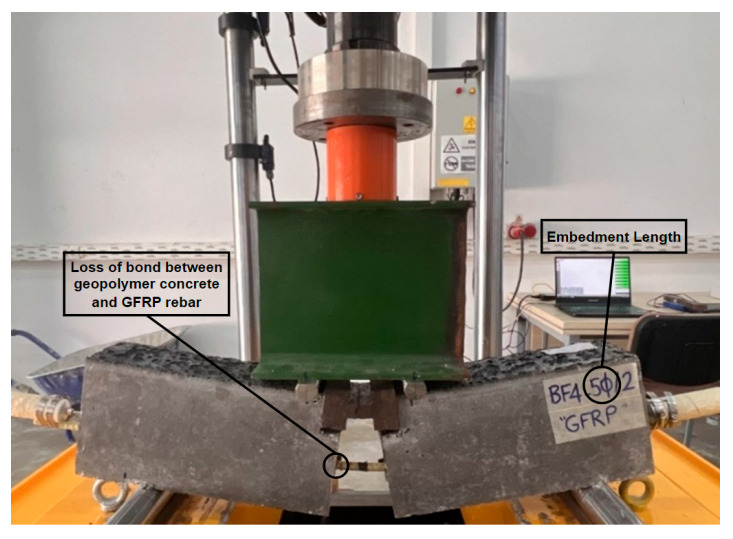
A specimen image after the hinged beam test.

**Figure 9:**
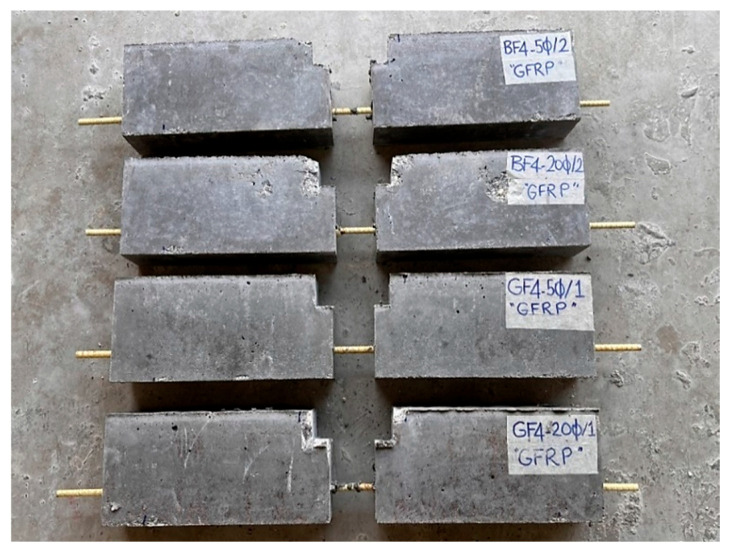
Post-test images of some test specimens.

The image of the specimen after the test, which ended with the stripping of the GFRP bar, is given in Figure 8. Post-test images of some test specimens are also shown in Figure 9. As can be seen from the figures, the GFRP bars in all specimens were subjected to stripping and reached their ultimate tensile stress with pull-out failure.

## 3. Results and Discussion

### 3.1. Preliminary Experiment Results

The flow diameter of fresh-state geopolymer concrete determined by a slump test [49] and the compressive strength and flexural tensile strength of hardened-state geopolymer concrete are given in Table 6.

Compared to plain geopolymer concrete (reference) specimens, the average compressive strength and tensile strength values of chopped glass fiber-added specimens increased by 9.4% and 87%, respectively. The compressive strength values of chopped basalt fiber-added specimens remained constant, while the tensile strength values increased by 62.7% compared to the reference specimens.

### 3.2. SEM Analysis of Geopolymer Concrete

**Figure 10:**
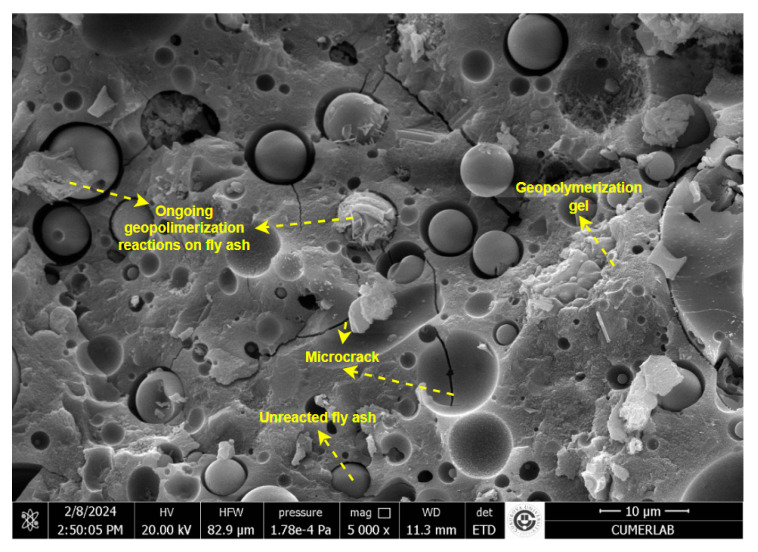
SEM image of geopolymer concrete sample cured at 100 °C.

Geopolymer mixes with Ms values of 1.1, 1.2, and 1.3 were first tested for workability, and the samples were subsequently cured at temperatures of 80 °C, 100 °C, and 120 °C. The geopolymer concrete mixture with a 1.2 MS modulus, cured at 100 °C, and exhibiting the desired compressive strength and adequate workability was selected for bond testing. Geopolymer concrete samples suitable for geopolymer beam production to be examined for bond performance were imaged using a Quanta 650 Field Emission SEM model scanning electron microscope (SEM) operating at 10 kV, Çukurova University, Adana, Turkey. Before analysis, the specimens were sputter-coated with a gold–palladium alloy. Figure 10 presents an SEM image of the selected hardened stage of the selected sample.

The SEM image seen in Figure 10 generally represents a binding matrix. When the matrix is examined, it is seen that there are ongoing geopolymerization reactions on the fly ash at different points. It is noteworthy that geopolymerization gels that have completed their transformation into a binding matrix have formed at some points. In addition, it is seen that there are some microcracks in the completed geopolymer gels. The ongoing and completed geopolymerization gels show that the binding matrix exists in the structure, and that the composite has gained a certain strength. The microcracks are located in some areas of the matrix in a way that negatively affects the strength. The SEM image shows that there are also fly ash grains that have not undergone any reaction in the matrix.

Geopolymerization initiates at the surface of the unactivated fly ash particles, which retain their spherical granular shape. Additionally, some microcracks are observed in the microstructure.

### 3.3. Bond-Slip Behavior of GFRP Rebar in Glass and Basalt Fiber-Reinforced Geopolymer Concrete

**Figure 11:**
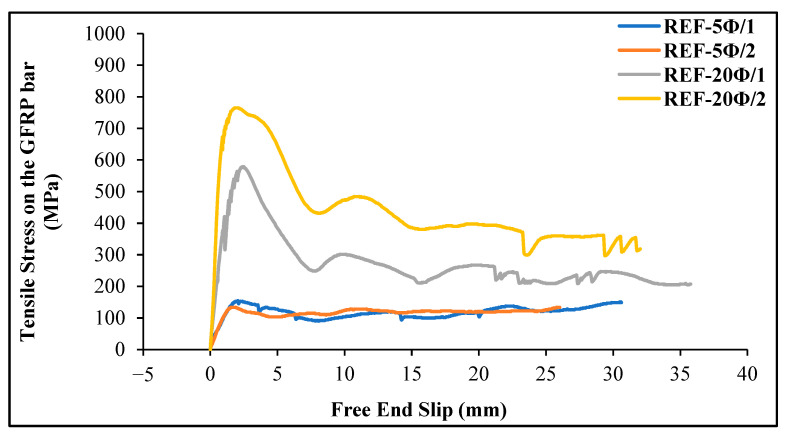
Tensile stress–slip curves of reference geopolymer concrete beams.

For each specimen, the variation of tensile stress on the GFRP bar is presented graphically. The graph in Figure 11 shows the tensile stress–slip curves for reference specimens (REF-5Ø and REF-20Ø) made of plain geopolymer concrete. For each mixture, two samples were taken and labeled as code 1 and code 2, as shown in Table 5.

For the reference specimens, it was observed that as the embedment length of the GFRP bar increased, the tensile stress also increased.

**Figure 12:**
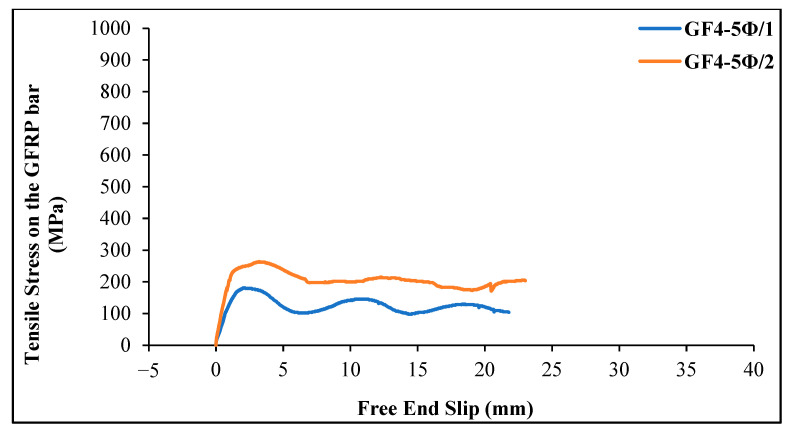
Tensile stress–slip curves of GFRP bars in glass fiber-reinforced geopolymer concrete beams (4 kg/m^3^ glass fiber and 5 Ø embedment length).

Figure 12 shows the tensile stress–slip curves of GFRP bars obtained from chopped glass fiber-reinforced geopolymer specimens (4 kg/m^3^) with an embedment length of 5 Ø = 60 mm. For the first beam specimen (GF4-5Ø/1), the first slip in the GFRP bar occurred at a stress value of 9.42 MPa. The maximum tensile stress value on the bar was 180.91 MPa, and the corresponding free-end slip value was determined as 2.16 mm. The maximum load on the bar was 20.46 kN, and the bond stress on the bar was calculated as 9.05 MPa. The final slip value was 21.77 mm. In the second beam specimen (GF4-5Ø/2), the first slip of the GFRP bar occurred at a stress value of 11.31 MPa. The maximum tensile stress recorded was 263.82 MPa, and the free-end slip at this stress was measured at 3.16 mm. The maximum load on the bar was 29.84 kN, and the bond stress of the bar was 13.19. The final slip value was 23.00 mm.

**Figure 13:**
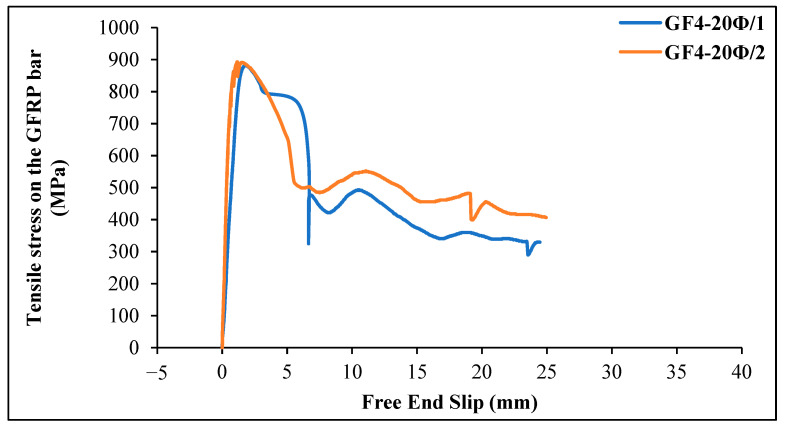
Tensile stress–slip curves of GFRP bars in glass fiber-reinforced geopolymer concrete beams (4 kg/m^3^ glass fiber and 20 Ø embedment length).

Figure 13 shows the tensile stress–slip curves of GFRP bars with an embedment length of 20 Ø = 240 mm and 4 kg/m^3^ chopped glass fiber content. The maximum tensile stress value on the bar for specimen 1 was 881.92 MPa, with a corresponding slip value of 1.84 mm, and the first slip in the GFRP bar occurred at a stress value of 20.73 MPa. The maximum load on the GFRP bar was 99.74 kN, and the bond stress of the bar was calculated as 11.02 MPa when the first slippage was observed. The final slip value was 24.45 mm. For specimen 2, the maximum tensile stress value on the bar was 893.22 MPa, with a corresponding slip value of 1.17 mm. As the loading continued, the slip increased rapidly, and the bond between the concrete and the GFRP bar was lost. The maximum load on the GFRP bar was 101.02 kN, and the bond stress of the bar was calculated as 11.17 MPa when the first slippage was observed. The final slip value was 24.98 mm.

**Figure 14:**
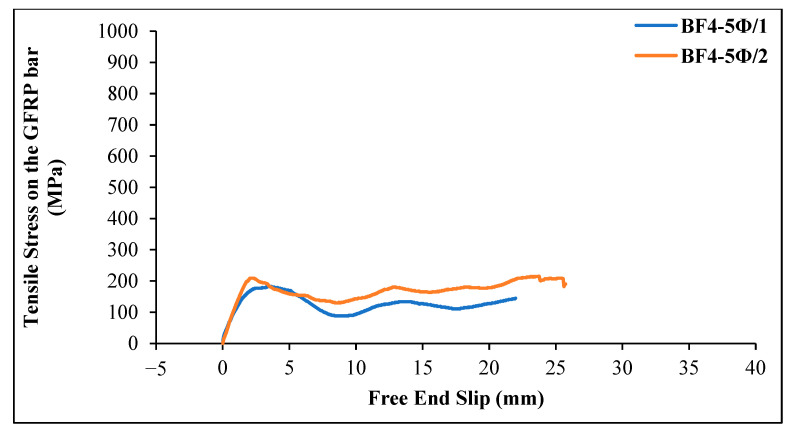
Tensile stress–slip curves of GFRP bars in basalt fiber-reinforced geopolymer concrete beams (4 kg/m^3^ basalt fiber and 5 Ø embedment length).

Figure 14 shows the tensile stress–slip curves of GFRP bars obtained from chopped basalt fiber-reinforced geopolymer specimens (4 kg/m^3^) with an embedment length of 5 Ø = 60 mm.

**Figure 15:**
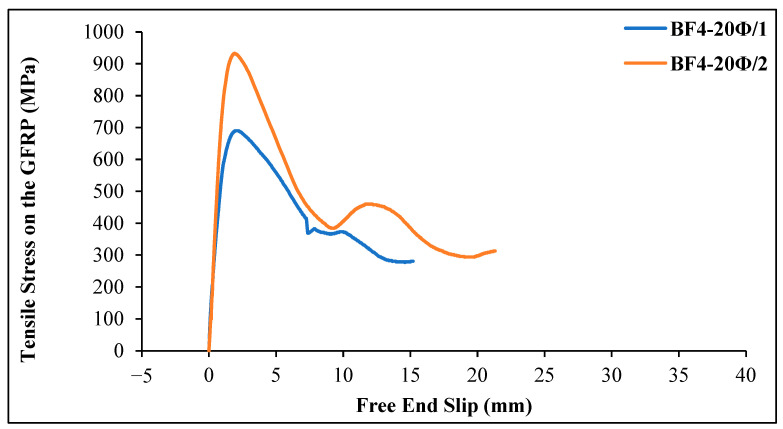
Tensile stress–slip curves of GFRP bars in basalt fiber-reinforced geopolymer concrete beams (4 kg/m^3^ basalt fiber and 20 Ø embedment length).

As can be seen from Figure 15, the first slip in the GFRP bar occurred at a stress value of 9.42 MPa in the basalt fiber-reinforced beam specimen. The maximum tensile stress value was 182.79 MPa, and the corresponding slip value was determined to be 3.64 mm when the first slippage was observed. The maximum load on the GFRP bar was 20.67 kN, and the bond stress of the bar was calculated as 9.14 MPa. For specimen 2, the first slip in the GFRP bar occurred at a stress value of 7.54 MPa. The maximum tensile stress value was 209.17 MPa, and the corresponding slip value was determined to be 2.32 mm. The maximum load on the GFRP bar was 23.67 kN, and the bond stress of the bar was calculated as 10.74 MPa.

Figure 15 shows the tensile stress–slip value changes for GFRP bars in chopped basalt fiber-added geopolymer concrete beam specimens 1 and 2 with an embedment length of 20 Ø = 240 mm. The maximum tensile stress value for specimen 1 was 689.70 MPa, with a corresponding slip value of 2.21 mm. The first slip in the GFRP bar occurred at a stress value of 13.19 MPa. The maximum load on the bar was 78.00 kN, and the bond stress of the bar was calculated as 8.62 MPa. The final slip value was 15.2 mm. For specimen 2, the maximum stress value was 932.80 MPa, with a corresponding slip value of 1.92 mm. As the loading continued, the slip increased rapidly, and the bond between the concrete and the bar was lost. The maximum load on the bar was 105.50 kN, and the bond stress of the GFRP bar was calculated as 11.66 MPa. The final slip value was 21.31 mm.

When comparing Figure 14 and Figure 15, the tensile stress values for specimens with a 20 Ø embedment length are higher than those for specimens with a 5 Ø embedment length.

**Figure 16:**
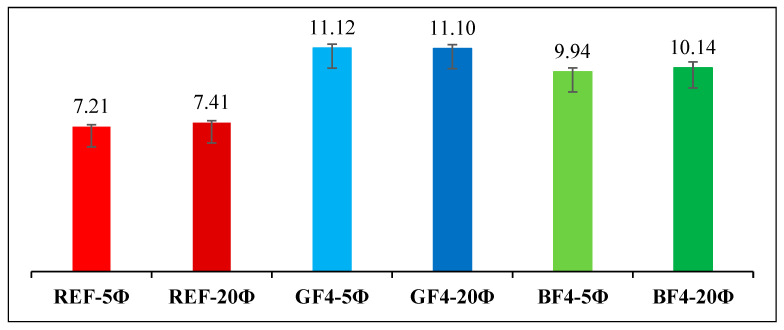
The average ultimate bond stress (τ_u_) values depending on the embedment length.

Figure 16 shows the variation of the average ultimate bond stress values depending on the embedment length. The average ultimate bond stress values increased by 54.2% and 49.8% with glass fiber addition for embedment lengths of 5 Ø and 20 Ø, respectively, and by 37.8% and 36.8% with basalt fiber addition compared to plain specimens, respectively. For all types of specimens, it can be concluded that increasing the embedment length does not significantly affect the average bond stress values. It can be also stated that the bond stress values for GFRP bars in chopped glass fiber-added beam specimens are higher than those for GFRP bars in chopped basalt fiber-added beam specimens.

**Figure 17:**
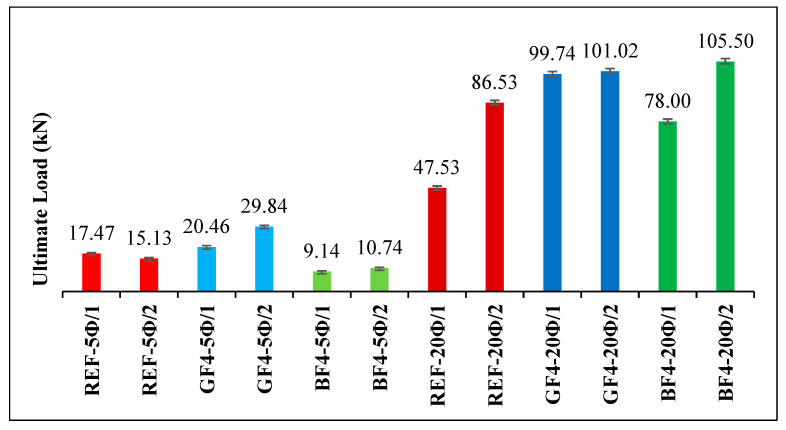
The ultimate load values on GFRP bars for basalt and glass fiber geopolymer specimens.

**Figure 18:**
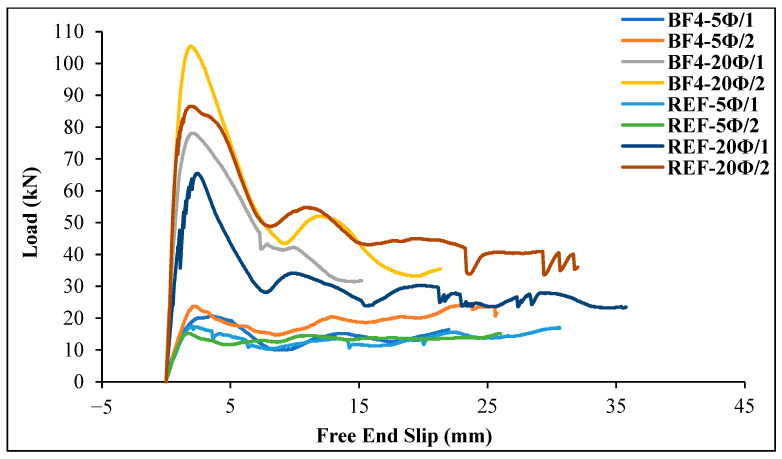
The load–slip curves for the basalt fiber-reinforced specimens compared to the reference specimens.

**Figure 19:**
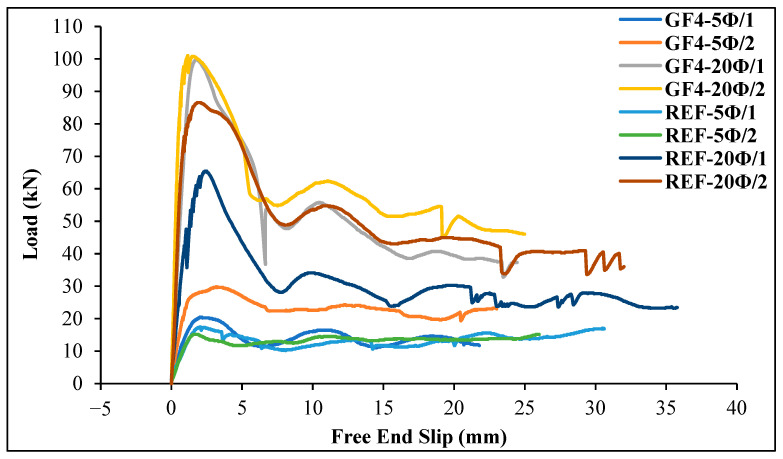
The load–slip curves for the glass fiber-reinforced specimens compared to the reference specimens.

Figure 17 shows the ultimate load values on GFRP bars in glass and basalt fiber-reinforced geopolymer specimens with 5 Ø and 20 Ø embedment lengths. Load–slip curves of the specimens were also obtained using the results given in Table 7, as shown in Figure 18 and Figure 19.

As can be seen from Table 7 and Figure 18 and Figure 19, the load values of the GFRP bar for chopped basalt or glass fiber-added geopolymer concrete beam specimens increase as the embedment length of the bar increases. As shown in Figure 18, for the basalt fiber-reinforced specimens, the ultimate load on the GFRP bar was reached in the 20 Ø embedment length specimen (BF4-20Ø/2). For this specimen, the ultimate load value of the GFRP bar increased by 21.82% compared to the reference specimen (REF-20Ø/2). Similarly, Figure 19 shows that for the glass fiber-reinforced specimens, the ultimate load on the GFRP bar occurred in the 20 Ø embedment length specimen (GF4-20Ø/2), and the ultimate load value on the GFRP bar for this specimen increased by 16.75% compared to the reference specimen (REF-20Ø/2).

**Figure 20:**
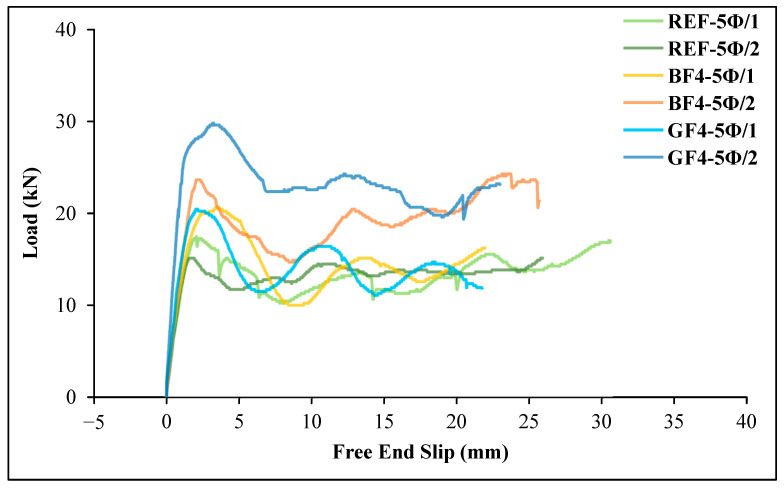
The load–slip curves of the specimens for 5 Ø embedment length.

**Figure 21:**
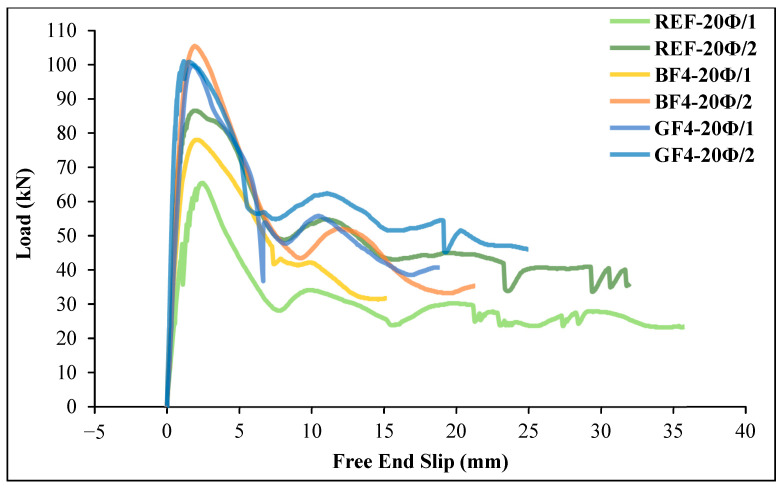
The load–slip curves of the specimens for 20 Ø embedment length.

Figure 20 and Figure 21 illustrate the effect of fiber type on bond strength for a fixed embedment length. For both embedment length conditions, the bond strengths of glass fiber specimens were higher than those of basalt fiber and non-fiber specimens. And also, these figures indicate that both fiber types have a positive effect on bond behavior.

Table 7 shows that all hinged beam specimens produced within the scope of the study resulted in pull-out failure, regardless of the embedment length. However, it is generally expected that for an embedment length of 20 Ø, the bond between the concrete and bars is expected to be better [43]. Albidah et al. [41] investigated the bond strength between geopolymer concrete and steel rebars and GFRP bars. As a result of the experiments, they found that the specimens with GFRP bars experienced pull-out failure. They also found that the bond strength of the steel rebars was higher than that of the GFRP bars. Mazaheripour et al. [24] reported that tensile failure occurred for all embedment lengths of 5 Ø, 10 Ø, and 20 Ø for specimens containing GFRP bars, and the bar surface had a significant effect on the bond stress. Gültekin et al., (2022) [50] evaluated the fresh properties and fracture energy of self-compacting concrete reinforced with basalt and glass fibers. They stated that fiber addition improves the bond behavior in composite systems. Kartal et al., (2020) [51] examined the load-deflection behavior of reinforced concrete beams reinforced with hybrid FRP-steel reinforcement and drew attention to the importance of bond properties on the load transfer mechanism in such systems. In these studies, the advantages of the reinforcement–matrix interface and hybrid systems were mentioned. Ertürkmen et al., (2025) [52] comprehensively examined the effect of glass and basalt fibers on the bond-slip behavior of steel reinforcement in environmentally friendly fly ash-based geopolymer concretes using the hinged beam method. This approach is an effective method used to measure the bond performance between the reinforcement and concrete, and it was found in the study that glass and basalt fibers improved the bond between the reinforcement and the matrix.

**Figure 22:**
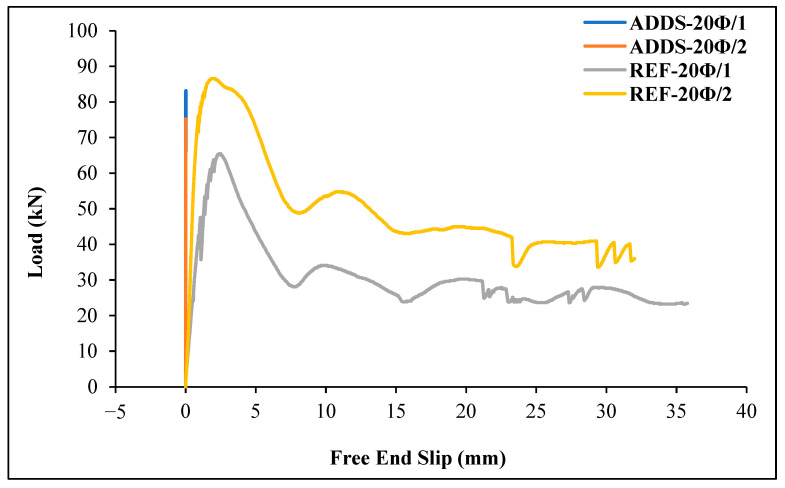
Comparison of load-free end slip curves between steel-reinforced additional reference specimens and GFRP-reinforced reference specimens with a 20 Ø embedment length.

The absence of studies in the literature on the bond between geopolymer concrete and GFRP bars, combined with the experimental results showing pull-out failure across different embedment lengths and fiber types, indicates the need to investigate whether the loss of bond is attributed to the concrete or the GFRP bar. To investigate this, additional beam specimens with steel rebar and a 20 Ø embedment length were produced using a geopolymer mixture without fibers. This was conducted to determine whether the pull-out failure observed in all the experiments was caused by the concrete or the surface of the rebar. The results obtained from additional beam specimens are presented in Table 8. A comparison of load–slip curves between additional specimens with steel rebar and reference plain geopolymer concrete beam specimens with GFRP bars with a 20 Ø embedment length is given in Figure 22.

As can be seen from Table 7 and Figure 22, when the steel rebar was used in the specimens, there was no loss of bond between the geopolymer concrete and the steel rebar until the bar rupture. This result reveals that the pull-out results of all experiments using GFRP bars are completely due to the bar, especially the surface of the rebar.

## 4. Conclusions

This study examined the bond performance of GFRP bars in hinged beam specimens made of geopolymer concrete, including chopped basalt or glass fibers, using a four-point loading test.

By using beam specimens, the bond between the GFRP bar and geopolymer concrete was clearly observed, and load–slip curves could be established. According to the findings, the following conclusions can be drawn.

- The ultimate load values of GFRP bars in fiber-reinforced geopolymer concrete beams increase with the embedment length, but the maximum bond stress is higher for glass fiber-reinforced geopolymer concrete compared to basalt fiber-reinforced concrete.
- Since all experiments resulted in the pull-out of GFRP bars for all types of specimens, no meaningful relations were found between the embedment length and the average bond stress values. However, it can be said that increasing the embedment length does not significantly affect the average bond stress values for all specimen types examined in the study.
- Glass fiber-reinforced geopolymer concrete showed 49% higher bond strength than geopolymer without fiber for samples with 20 Ø embedment length, while basalt fiber-reinforced geopolymer concrete showed a 37% increase. This means that glass fiber was found to be more effective than basalt fiber in improving the bond strength of GFRP geopolymer.
- The study suggests that the surface type of GFRP bars plays a key role in bond performance, especially at the 20 Ø embedment length.
- The bond performance of FRP bars is complex, and findings from this study contribute to the ongoing debate in the field.

## Figures and Tables

**Table 1:** The physical properties of the aggregates.

Aggregate	Density (kg/m^3^) (STD)	Water Absorption (%) (STD)	Stock Humidity (%) (STD)
Coarse (4.75–12.7 mm)	2670 (0.82)	1.11 (0.01)	0.15 (0.01)
Fine (0–4.75 mm)	2660 (1.89)	2.64 (0.03)	0.24 (0.01)

**Table 2:** The chemical composition of fly ash.

Chemical Composition	(%)
Al_2_O_3_	21.00
SiO_2_	53.00
SO_3_	1.50
K_2_O	2.70
CaO	4.95
TiO_2_	1.31
Fe_2_O_3_	13.83
Na_2_O	0.69
MgO	0.09
Others	0.93
Loss on Ignition	1.32

**Table 3:** Geopolymer concrete mix.

Ms	Materials (kg/m^3^)				Dry Mixture	Activator
	Fly Ash	Aggregate	Sodium Silicate (Na_2_SiO_3_)	Sodium Hydroxide (NaOH)		
**1.2**	497.81	1493.43	270.92	27.88	1991.20	298.80

**Table 4:** Some mechanical properties of GFRP bars used in the bond tests.

Properties	Values
**Tensile strength**	800–1300 MPa
**Modulus of elasticity**	55,000 MPa

**Table 5:** Coding details of all hinged beam specimens.

Specimen Code Name	Fiber Type	Fiber Length	Fiber Amount	Embedment Length
**REF-5Ø/1 (GFRP)** **REF-5Ø/2 (GFRP)**	Non- Fiber	-	-	5 Ø
**REF-20Ø/1 (GFRP)** **REF-20Ø/2 (GFRP)**				20 Ø
**GF4-5Ø/1 (GFRP)** **GF4-5Ø/2 (GFRP)**	Chopped Glass	6 mm	4 kg/m^3^	5 Ø
**GF4-20Ø/1 (GFRP)** **GF4-20Ø/2 (GFRP)**				20 Ø
**BF4-5Ø/1 (GFRP)** **BF4-5Ø/2 (GFRP)**	Chopped Basalt	6 mm	4 kg/m^3^	5 Ø
**BF4-20Ø/1 (GFRP)** **BF4-20Ø/2 (GFRP)**				20 Ø

**Table 6:** Compressive strength, flexural tensile strength, and flow diameters of the cubic and prismatic geopolymer concrete samples.

Samples	Compressive Strength (MPa) (STD)	Flexural Tensile Strength (MPa)	Flow Diameter (cm)
**REF**	56.53 (1.55)	3.54 (0.07)	57 (0.25)
**GF4**	61.84 (2.05)	6.62 (0.11)	46 (0.5)
**BF4**	56.39 (1.82)	5.76 (0.08)	43 (0.5)

**Table 7:** Bond test results for all specimens under hinged beam conditions.

Specimen Code	Ultimate Load (kN)	Average Ultimate Load (kN)	Ultimate Stress on Rebar (MPa)	Average Ultimate Stress on Rebar (MPa)	Ultimate Bond Stress (MPa)	Average Bond Stress (MPa)	Slip at Ultimate Stress (mm)	Average Slip at Ultimate Stress (mm)	First Slip Stress (MPa)	Failure Type
**REF-5Ø/1**	17.47	16.30	154.52	144.16	7.73	7.21	2.05	1.98	9.42	Pull-out
**REF-5Ø/2**	15.13		133.80		6.69		1.90		5.65	
**REF-20Ø/1**	47.53	67.03	420.23	592.66	5.25	7.40	1.05	1.57	11.31	Pull-out
**REF-20Ø/2**	86.53		765.08		9.56		2.08		11.31	
**GF4-5Ø/1**	20.46	25.15	180.91	222.37	9.05	11.12	2.16	2.66	9.42	Pull-out
**GF4-5Ø/2**	29.84		263.82		13.19		3.16		11.31	
**GF4-20Ø/1**	99.74	100.38	881.92	887.57	11.02	11.09	1.84	1.51	20.72	Pull-out
**GF4-20Ø/2**	101.02		893.22		11.17		1.17		28.27	
**BF4-5Ø/1**	20.67	22.17	182.79	195.98	9.14	9.94	3.64	2.98	9.42	Pull-out
**BF4-5Ø/2**	23.67		209.17		10.74		2.32		7.54	
**BF4-20Ø/1**	78.00	91.75	689.70	811.25	8.62	10.14	2.21	2.07	13.19	Pull-out
**BF4-20Ø/2**	105.5		932.80		11.66		1.92		13.19	

**Table 8:** Bond test results for additional specimens with steel rebar and 20 Ø embedment length.

Specimen Code	Ultimate Load (kN)	Average Ultimate Load (kN)	Ultimate Stress on Rebar (MPa)	Average Ultimate Stress on Rebar (MPa)	Ultimate Bond Stress (MPa)	Average Bond Stress (MPa)	Slip at Ultimate Stress (mm)	Average Slip at Ultimate Stress (mm)	First Slip Stress (MPa)	Failure Mode
**ADDS-20Ø/1**	83.12	79.175	734.93	700.070	9.19	8.755	0	0	712.32	Bar rupture
**ADDS-20Ø/2**	75.23		665.21		8.32		0		665.21	

## Data Availability

The original contributions presented in this study are included in the article. Further inquiries can be directed to the corresponding author.

## Abbreviations

The following abbreviations are used in this manuscript:

FRP	Fiber-reinforced polymer
AFRP	Aramid fiber-reinforced polymer
GFRP	Glass fiber-reinforced polymer
CFRP	Carbon fiber-reinforced polymer
BFRP	Basalt fiber-reinforced polymer
HFRP	Hybrid fiber-reinforced polymer
